# Comment on “Effects of a Mind-Body Medicine Group Program for Cancer Patients: A Retrospective Cohort Study”

**DOI:** 10.1177/15347354251398038

**Published:** 2025-11-19

**Authors:** Chutharat Thanchonnang, Nathkapach K. Rattanapitoon, Nav La, Schawanya K. Rattanapitoon

Dear Editor,

We read with great interest the recent article by Schricker et al.^1^ The authors should be commended for addressing an underexplored yet clinically pivotal aspect of integrative oncology—structured mind-body interventions embedded within conventional cancer care. Their findings, showing significant improvements in fatigue, quality of life, and emotional well-being, provide valuable evidence supporting the psychophysiological benefits of such programs. We would, however, like to offer several reflections and propose directions that could strengthen future translational research in this field.

The retrospective nature of the study limits causal inference, yet the reported association between baseline sleep quality and treatment responsiveness is highly intriguing. This observation may reflect an underlying *neuroimmune “responder phenotype”*—patients whose dysregulated sleep-wake cycles mirror altered hypothalamic–pituitary–adrenal (HPA) axis activity and proinflammatory signaling.^2^ Incorporating objective biomarkers such as diurnal cortisol slope, heart rate variability, or circulating cytokines (eg, IL-6, CRP) in prospective designs could help elucidate whether improvements in sleep act as a mechanistic conduit for the broader psychological and physical benefits of mind-body interventions.

The cohort’s predominance of women with breast cancer reiterates a persistent gender imbalance observed across mind-body medicine research.^3^ Beyond recruitment logistics, this disparity may arise from gendered differences in emotional expressivity, social support utilization, and health-seeking behavior. Integrative oncology would benefit from examining whether sex-linked neuroendocrine factors—such as oxytocinergic tone or stress reactivity—modulate responsiveness to these interventions. Such inquiry would move the field toward *sex-informed precision mind-body medicine.*

The finding that patients further from diagnosis derived greater benefit invites consideration of *therapeutic timing.* Early in the cancer trajectory, patients are often cognitively overloaded and emotionally saturated, reducing receptivity to introspective practices.^4^ A staggered, phase-specific model—delivering low-intensity stress-management modules during active treatment followed by immersive programs during survivorship—could optimize neuroplasticity, adherence, and psychological readiness.

Translating within-group benefits into real-world oncology pathways remains an urgent priority. Embedding mind-body medicine (MBM) programs into institutional survivorship frameworks and linking participation to measurable endpoints—such as reduced unplanned admissions, improved immune recovery, or attenuated inflammation—could demonstrate tangible healthcare value. Digital or hybrid delivery models, augmented by wearable sleep and stress tracking, may further enable equitable, scalable implementation.

Alignment with standardized patient-reported outcomes recommended by the Society for Integrative Oncology–ASCO guidelines^5^ would facilitate cross-study comparability and evidence harmonization. A next-generation trial design could integrate psychometric, physiologic, and biomarker endpoints to construct a *biopsychosocial response map*—a precision framework capable of predicting which patients will benefit most, when, and why.

In summary, Schricker et al provide an important foundation for advancing mind-body oncology from a supportive-care adjunct toward a mechanism-based, precision discipline. Future studies that integrate sleep physiology, neuroimmune profiling, and digital phenotyping will not only clarify causal pathways but may also enable *personalized mind-body prescriptions*—an evolution that could redefine how psychosocial resilience is cultivated in cancer survivorship.


Chutharat Thanchonnang, PhD *Faculty of Public Health, Mahidol University, Nakhon Sawan Campus, Thailand*
Nathkapach K. Rattanapitoon, PhD *FMC Medical Center, Nakhon Ratchasima, Thailand*
Nav La, MD, PhD *Faculty of Medicine, International University, Phnom Penh, Cambodia*
Schawanya K. Rattanapitoon, MD, FRCFPT *FMC Medical Center, Nakhon Ratchasima, Thailand*

